# Precocious Alterations of Brain Oscillatory Activity in Alzheimer’s Disease: A Window of Opportunity for Early Diagnosis and Treatment

**DOI:** 10.3389/fncel.2015.00491

**Published:** 2015-12-21

**Authors:** Valentine Hamm, Céline Héraud, Jean-Christophe Cassel, Chantal Mathis, Romain Goutagny

**Affiliations:** ^1^Centre National de la Recherche Scientifique UMR 7364, Laboratoire de Neurosciences Cognitives et AdaptativesStrasbourg, France; ^2^Université de Strasbourg, Neuropôle de StrasbourgStrasbourg, France

**Keywords:** Alzheimer’s disease, mild cognitive impairment, recognition memory, spatial navigation memory, oscillatory activity, electroencephalography

## Abstract

Alzheimer’s disease (AD) is the most common form of neurodegenerative dementia accounting for 50–80% of all age-related dementia. This pathology is characterized by the progressive and irreversible alteration of cognitive functions, such as memory, leading inexorably to the loss of autonomy for patients with AD. The pathology is linked with aging and occurs most commonly around 65 years old. Its prevalence (5% over 65 years of age and 20% after 80 years) constitutes an economic and social burden for AD patients and their family. At the present, there is still no cure for AD, actual treatments being moderately effective only in early stages of the pathology. A lot of efforts have been deployed with the aim of defining new AD biomarkers. Successful early detection of mild cognitive impairment (MCI) linked to AD requires the identification of biomarkers capable of distinguishing individuals with early stages of AD from other pathologies impacting cognition such as depression. In this article, we will review recent evidence suggesting that electroencephalographic (EEG) recordings, coupled with behavioral assessments, could be a useful approach and easily implementable for a precocious detection of AD.

## Introduction

Alzheimer’s disease (AD) is a progressive neurodegenerative disorder that starts with mild short-term memory deficits before progressively culminating in total loss of cognitive and executive functions. Currently, the precise etiology of the pathology is not known and there is no cure. Genetic studies (Price et al., 1998; Van Cauwenberghe et al., 2015) have identified mutations in the genes of the transmembrane amyloid peptide precursor protein (APP) and those of presenilins 1 and 2 (PS1, PS2) responsible for rare dominantly inherited early onset familial AD (FAD). Proteolytic processing of APP first by the β-site APP cleaving enzyme, followed by the PS-containing γ-secretase complex, generates amyloid-β (Aβ) peptides that deposit in amyloid plaques. Many studies showed an increased production of more amyloidogenic Aβ peptides associated with FAD-linked mutations, providing strong support for the amyloid hypothesis (Hardy and Selkoe, 2002). According to this conceptual framework, it would be the early accumulation of soluble Aβ in specific brain areas that elicits abnormal patterns of neuronal activity leading to cognitive decline (Palop and Mucke, 2010). Therefore, a lot of efforts have been deployed in order to lower Aβ levels as a possible therapy for AD. Various types of treatments have been tested including the use of γ-secretase inhibitors and immunization against Aβ. Unfortunately, these drugs were less successful than expected, inducing no improvement, or even a worsening of cognitive functions, often accompanied by drastic side-effects that preclude their use as suitable therapeutics for AD (Mikulca et al., 2014). Diverse hypothesis were put forward to explain the disappointing results obtained by current anti-Aβ treatments. First, other APP fragments, like the β-carboxy terminal fragment (β-CTF; Lahiri et al., 2002; Pimplikar et al., 2010; Lauritzen et al., 2012; Tamayev et al., 2012; Goutagny et al., 2013), the amyloid intracellular domain (AICD), or the recently described CTF-η (Willem et al., 2015), might play key roles in AD pathogenesis and associated cognitive symptoms. A second hypothesis highlights the fact that treatments were given too late in the time-course of AD, when neuronal damages are already too extensive and irreversible. However, these two points are closely linked. We need to develop new early biomarkers, independent of the amyloid hypothesis, that do not entirely rely on Aβ dosage. In this article, we will review recent evidences indicating that the characterization of oscillatory activity in patients using electroencephalographic (EEG) recordings, a cost-efficient and easily implementable strategy, might represent a new opportunity for the early detection of AD. In addition, we propose that characterization of cross-frequency coupling (CFC), a specific motif of oscillatory interactions, might represent an extremely sensitive early biomarker.

## Brain Oscillatory Activity

Cognitive processes (i.e., information processing and storage by brain networks) require a highly coordinated operation of multiple neuronal groups. One likely mechanism is through the coordinated rhythmic activity of neuronal populations, which give rise to oscillations (Womelsdorf et al., 2007). These oscillations can be recorded using various techniques, such as electrocorticography, local field potential, magnetoencephalography, or EEG. Rhythmic fluctuations of electric potentials measured by these technics are generated by the spatial summation of highly synchronized post-synaptic potentials occurring in large clusters of neurons. They have an excellent temporal resolution (in the millisecond timescale) and now, new analytic methods allow locating generators of these oscillations with a decent spatial resolution even using EEG (around one voxel with the LORETA method in human EEG; Gianotti et al., 2007).

The spectral content of EEG is classically divided in five frequency bands: δ (from 1 to 4 Hz), θ (4 to 7 Hz), α (8 to 12 Hz), β (15 to 30 Hz), and γ (>30 Hz). On a functional level, these diverse oscillations are associated with different brain states. Oscillatory activities are related to global states (i.e., δ waves are mainly present during sleep) or specific behaviors (i.e., β rhythm is usually associated to motor tasks and is thought to reflect the activity of motor cortices; Pfurtscheller et al., 1998). Some frequency domains are more closely related to cognitive processes. First, θ oscillations are thought to play a key role in working memory processes (Sauseng et al., 2010). θ phase affects memory processing through the modulation of neuronal plasticity within hippocampal and cortical areas and plays a modulatory role in the induction of long-term-potentiation (LTP), a long lasting enhancement of synaptic efficacy which may constitute one of the cellular substrates for learning and memory. In addition, θ rhythm is also strongly linked to hippocampal pyramidal cells that code for spatiotemporal aspects of the animal’s environment (Huxter et al., 2003). Second, α oscillations are more related to attentional processes by filtering out irrelevant informations and preventing interference from conflicting stimuli (Klimesch, 2012). Finally, γ oscillations are modulated by a variety of cognitive processes such as object recognition and working memory (Tiitinen et al., 1993; Yordanova et al., 1997a,b; Herrmann and Mecklinger, 2000; Fries et al., 2001; Debener et al., 2003; Herrmann et al., 2004a,b) and are thought to temporally link distributed cell assemblies from different sources that are processing related informations.

Slow and fast oscillatory activities are not independent. Indeed, slow and fast rhythms interact with each other, the phase of slow oscillations (mainly θ rhythm) being able to modulate the amplitude of fast oscillations (β and γ rhythms). This phenomenon, known as CFC, is positively associated with cognitive processes in humans (Canolty et al., 2006; Händel and Haarmeier, 2009; Axmacher et al., 2010), monkeys (Canolty et al., 2010), rats (Tort et al., 2008, 2009) and mice (Wulff et al., 2009). More specifically, it is hypothesized that at fast frequencies, CFC would allow distributed brain regions to be synchronized (using the slow one as a “carrier”), which consequently facilitates communication.

Given the key role played by oscillatory activities on cognitive processes such as memory, numerous studies have closely looked at brain oscillatory alterations in AD patients, as well as in animal models of the pathology. In the next parts of this article, we will review recent findings on oscillatory activity alterations in the time course of AD.

## Oscillatory Activity in Mild Cognitive Impairment (MCI) and AD Patients

More than 980 articles in the last 40 years have looked at EEG activity in mild cognitive impairment (MCI) and/or AD patients. Indeed, with the development of new analytic methods that can account for different confounding results (for example, volume conduction), EEG activity seems sensitive enough for an early detection of preclinical AD and predictive of future conversion from MCI to AD.

The majority of studies focusing on EEG characterization in AD patients have been done using resting state paradigms. Resting state EEG corresponds to recordings performed in the motionless subject with eyes closed. This task is a fully standardized procedure and can therefore be done in highly comparable experimental conditions. Compared to age-matched healthy control subjects, both MCI and AD patients exhibit an increase in relative power of slow oscillations (δ and θ rhythms) associated with a decrease in relative power of fast oscillations (α, β, and γ rhythms; van der Hiele et al., 2007; Czigler et al., 2008; Moretti et al., 2010). The relative amplitude of θ oscillations has been proposed as a marker for AD as it allows the correct classification of 85% of MCI subjects, distinguishing the ones who progress to clinically manifested AD from those who remain stable (Jelic et al., 2000). Furthermore, increased θ power is already present in subjects with subjective complaints 7 years before decline to the MCI state (Prichep et al., 2006).

However, differences in resting state EEG might not be specific to AD. Indeed, multiple types of dementia could also be characterized by similar global network alterations. As an example, an increase in relative θ power is also found in dementia with Lewy bodies (Kai et al., 2005) and a decrease in relative γ power is also found in normal aging, after a brain injury or a stroke (Herrmann and Demiralp, 2005).

Therefore, and to achieve higher specificity, it could be suitable to combine behavioral paradigms in real time with electrophysiological recordings. During behavioral tasks, memory-related activation reveals specific EEG functional differences between MCI patients and control ones that facilitates the early diagnostic of probable AD. As an example, haptic tasks are sensitive to early perceptive-cognitive and functional deficits in MCI patients. Indeed, during tactile tasks, θ-power over right occipital regions is a suitable marker to distinguish healthy subjects from MCI patients (Grunwald et al., 2002). In addition, in a face-name encoding mnemonic task, the recording of EEG alterations is associated with the Mini-Mental State Examination and may serve as a clinically valuable marker for disease severity (Garn et al., 2014).

However, multiple forms of memory are affected in AD (Didic et al., 2011) and episodic memory impairment is not specific to AD but is also found in other types of dementia and psychiatric disorders. It was proposed that navigation deficits could help to distinguish patients at higher risk of developing AD from individuals with normal cognitive aging and those with other neurodegenerative diseases (Lithfous et al., 2013). Indeed, spatial disorientation is already present at MCI and early AD stages. Specific spatial tasks in both virtual or real world paradigms may possibly predict the conversion from normal aging to MCI and from MCI to dementia (Kalová et al., 2005; Laczó et al., 2011; Weniger et al., 2011; Moodley et al., 2015). Spatial navigation depends on θ (Cornwell et al., 2008; Jacobs et al., 2010; Snider et al., 2013) and γ (Park et al., 2014) oscillations. Therefore, behavioral assessments of spatial memory processes combined with EEG techniques might represent a promising strategy for an early detection of preclinical AD with a high specificity.

## Oscillatory Activity in Animal Models of AD

In spite of the considerable restriction that few spontaneous animal models recapitulate the entire spectrum of the sporadic form of AD (Strittmatter et al., 1993; Giannakopoulos et al., 1997; Inestrosa et al., 2005; Bons et al., 2006; Toledano et al., 2014; Stefanova et al., 2014, 2015), parallel research on animals has provided an essential contribution in understanding the mechanisms underlying abnormal oscillatory patterns in AD.

Hippocampal slices preparations from rodents and transgenic mice models of AD constitutes a useful tool for investigating mammalian synaptic alterations during amyloid pathology (Mathis et al., 2011; Hazra et al., 2013). However, spontaneous oscillations are not present in hippocampal slices. Indeed, hippocampal oscillatory activity is the product of multiple intra- and extra-hippocampal oscillators (the hippocampus and the medial septum for θ oscillations; Borisyuk et al., 1999; Denham and Borisyuk, 2000; Wang, 2002; Goutagny et al., 2009) and an intra-hippocampal excitation/inhibition loop, together with inputs from the entorhinal cortex for γ rhythms (Bragin et al., 1995). The study of hippocampal oscillations in slices requires the application of a cholinergic or glutamatergic (kainate receptor) agonist to increase cellular excitability. With such a type of approach, it was shown that application of Aβ_1-42_ reduced the power of kainate-induced γ oscillations in mice (Kurudenkandy et al., 2014). However, the utility of these models must be considered in light of how well they mimic the actual phenomenon. A bath application of carbachol to hippocampal slices can generate either θ or γ rhythms depending on experimental parameters such as: slice orientation, thickness, drug concentration, and temperature (Konopacki et al., 1987; Fisahn et al., 1998; Fellous and Sejnowski, 2000). However, there is no evidence that in freely moving animals θ and γ rhythms require cholinergic neurotransmission. Another popular model of hippocampal γ rhythms uses kainite receptor activation (Traub et al., 2005). Once again, although robust γ can be observed in hippocampal preparations, it does not appear as though *in vivo* γ rhythms are mediated by kainate receptors. Only one report has generated a model of simultaneous θ and γ rhythms in the presence of kainic acid (Gloveli et al., 2005) which required a unique hippocampal slice containing transverse and longitudinal circuitries. The recent development of a new *in vitro* preparation, which respects the complex three-dimensional organization of intrinsic hippocampal circuits, has circumvented most of the issues aforementioned. Using this preparation, it is possible to characterize spontaneously occurring θ (Goutagny et al., 2009) and γ (Jackson et al., 2011) oscillations. In a transgenic mouse model of AD, the TgCRND8 mice, hippocampal θ-γ uncoupling was shown to precede soluble Aβ and plaque accumulation (Goutagny et al., 2013).

Oscillatory activities can also be measured in anesthetized animals (Xu et al., 2015). In this paradigm, θ oscillations can be recorded under urethane anesthesia either spontaneously or after sensory stimulation (tail or paw pinches) or electric stimulation of the brainstem *nucleus pontis oralis* (Bland and Whishaw, 1976). With this approach, it was shown that APP/PS1 transgenic mice showed an age-dependent decrease in hippocampal θ activity correlating with plaque load (Scott et al., 2012). However, under urethane anesthesia, θ oscillations are exclusively of type II (atropine-sensitive) and no type I θ (atropine-resistant) is present (Kramis et al., 1975). Therefore, in order to fully capture possible alterations in hippocampal oscillatory activity in animal models of AD, recordings in freely moving animals are required.

Many cognitive paradigms used in MCI and early AD diagnoses are based on verbal episodic memory tasks that present a translational problem for animal studies. Indeed, episodic memory, which is characterized by conscious recollection of context-rich events, is rather difficult to probe in animals. Several episodic-memory-like paradigms are currently being developed in rodents and apes but their extrapolation and dependence on a similar set of temporal lobe structures than human episodic memory still need to be confirmed. Fortunately, nature knows best, the hippocampus and the parahippocampal formation, responsible for episodic memory in humans, seem to have anatomical and functional homologs across mammal species. As an example, these brain regions are implicated in the encoding and retrieval of information related to environment during spatial navigation in rodents (Molter et al., 2012). In a recent study, rhesus monkeys learned how to freely drive a wheelchair to navigate through a complex maze, providing a strong support for an electrophysiological investigation of spatial navigation in the real world (Etienne et al., 2014). In rodents as in humans, spatial representations are related to modulation of θ oscillations as well as θ-γ coupling (Huxter et al., 2003; Bott et al., 2015). Interestingly, in a transgenic mouse model of AD, the Tg5xFAD mice, a decrease of θ and γ frequencies precedes disturbances in learning performances in a navigation task (Schneider et al., 2014).

## Conclusion

To conclude, with the support of the previously described results obtained both on animal models and patients, we propose that CFC alterations might constitute a promising early biomarker of AD. Indeed, modifications in hippocampal θ-γ coupling during spatial navigation might occur in the very first stages of AD and serve as a possible predictor for the pathology (Goutagny and Krantic, 2013). Future research aimed at identifying biomarkers based on combined EEG and behavioral testing approaches should integrate the fact that spatial navigation memory tasks used to diagnose AD in patients can be transposed to animals. In this way, animal studies leave the door open on diagnostic and therapeutic pathways that could be transposable in patients. Moreover, independently of progress made on earlier disease targets, it may be assumed that patients diagnosed at the most precocious stage of the pathology still have enough brain plasticity resources to sustain effective responses to therapeutic interventions, including environmental enrichment (Verret et al., 2013; Yeung et al., 2015), to stop the progression of AD or even reverse it.

## Author Contributions

VH and RG wrote the review. CM provided critical inputs. CH and JC helped to correct the manuscript.

## Conflict of Interest Statement

The authors declare that the research was conducted in the absence of any commercial or financial relationships that could be construed as a potential conflict of interest.
